# Complexities of retention in primary care randomised trials: A thematic analysis of in-depth interviews

**DOI:** 10.1186/1745-6215-12-S1-A132

**Published:** 2011-12-13

**Authors:** Valerie Brueton, Fiona Stevenson, Claire Vale, Greta Rait

**Affiliations:** 1 MRC General Practice Research Framework, London, WC2B 6NH, UK; 2 UCL Research Department Primary Care and Population Health, London, NW3 2FP, UK; 3 MRC Clinical Trials Unit, London, WC2B 6NH, UK

## Introduction

Loss to follow-up in randomised trials can cause bias, compromise study power, and affect the generalisability and reliability of results [1]. Many strategies are used to try to retain participants, however little is known about trialists’ experiences of implementing such strategies, or their perspectives of effectiveness. The complexity of these experiences and perceptions may influence the type of strategies used in different disease areas and with different population groups. We explored factors that trialists think contribute to loss to follow-up in primary care randomised trials, and whether some strategies to improve retention are perceived to be more successful than others.

## Methods

29 purposively sampled UK trialists including principal investigators n=10, research nurses n=9, and trial managers n=10 were invited for an in depth interview. Trialists were sampled from randomised trials conducted in UK primary care settings and published between 2000-2010. Randomised trials with high (>20%), moderate (5-20%) and low (<5%) rates of attrition were included in the sampling frame [2]. In-depth interviews were digitally recorded, transcribed verbatim, and anonymised. Concurrent thematic analysis was conducted. ATLAS ti 6.1 was used to organise and explore coded transcripts. Themes around each category were verified and confirmed by constant comparison and searching across all interviews for similar themes and categories for analysis.

## Results

29 in-depth interviews were conducted with 10 principal investigators, 10 trial managers, and 9 research nurses from primary care randomised trials in mental health, nutrition, elderly care, and chronic diseases. A major theme emerging across all interviews is the importance of communication between participant and trialist. Factors thought to contribute to retention include: rapport between participant and trialist, participant altruism, and flexibility around appointment schedules. Giving information about what the trial involves at the initial recruitment visit was considered to influence retention. Reducing burdens, both financial and physical, by provision of transport and reimbursement of costs were also considered useful.

## Conclusions

The findings provide a deeper understanding of the complexity of retention in randomised trials and may inform trialists’ choice of potentially effective strategies in future trials. In combination with an ongoing systematic review of randomised trials of retention strategies, we will highlight strategies or combinations of strategies that should be evaluated prospectively.
